# Homopharma: A new concept for exploring the molecular binding mechanisms and drug repurposing

**DOI:** 10.1186/1471-2164-15-S9-S8

**Published:** 2014-12-08

**Authors:** Yi-Yuan Chiu, Jen-Hu Tseng, Kuan-Hsiu Liu, Chih-Ta Lin, Kai-Cheng Hsu, Jinn-Moon Yang

**Affiliations:** 1 Institute of Bioinformatics and Systems Biology, National Chiao Tung University, Hsinchu, 30050, Taiwan; 2 Department of Biological Science and Technology, National Chiao Tung University, 75 Po-Ai Street, Hsinchu, 30050, Taiwan

## Abstract

**Background:**

Drugs that simultaneously target multiple proteins often improve efficacy, particularly in the treatment of complex diseases such as cancers and central nervous system disorders. Many approaches have been proposed to identify the potential targets of a drug. Recently, we have introduced Space-Related Pharmamotif (SRPmotif) method to recognize the proteins that share similar binding environments. In addition, compounds with similar topology may bind to similar proteins and have similar protein-compound interactions. However, few studies have focused on exploring the relationships between binding environments and protein-compound interactions, which is important for understanding molecular binding mechanisms and helpful to be used in discovering drug repurposing.

**Results:**

In this study, we propose a new concept of "Homopharma", combining similar binding environments and protein-compound interaction profiles, to explore the molecular binding mechanisms and drug repurposing. A Homopharma consists of a set of proteins which have the conserved binding environment and a set of compounds that share similar structures and functional groups. These proteins and compounds present conserved interactions and similar physicochemical properties. Therefore, these compounds are often able to inhibit the proteins in a Homopharma. Our experimental results show that the proteins and compounds in a Homopharma often have similar protein-compound interactions, comprising conserved specific residues and functional sites. Based on the Homopharma concept, we selected four flavonoid derivatives and 32 human protein kinases for enzymatic profiling. Among these 128 bioassays, the IC_50 _of 56 and 25 flavonoid-kinase inhibitions are less than 10 μM and 1 μM, respectively. Furthermore, these experimental results suggest that these flavonoids can be used as anticancer compounds, such as oral and colorectal cancer drugs.

**Conclusions:**

The experimental results show that the Homopharma is useful for identifying key binding environments of proteins and compounds and discovering new inhibitory effects. We believe that the Homopharma concept can have the potential for understanding molecular binding mechanisms and providing new clues for drug development.

## Background

Developing a new drug is difficult and takes on average of 13 years as well as US$1.8 billion [1]. Traditional approaches for rational drug design used the "one gene, one drug, one disease" paradigm to design exquisitely selective ligands of a single disease target. For example, gefitinib (trade name Iressa) and imatinib (trade name Gleevec) have been developed by this strategy and used for lung cancer and chronic myeloid leukemia, respectively. However, many drugs have been indicated that they can interact with more than one target protein [2-4]. Some unexpected efficacy can be linked to activity against additional targets, such as imatinib and PDGF receptor [5]. Previous work has identified that a drug interacts with 6 targets on average by analyzing a drug-target network consisting of 802 drugs and 4,746 interactions [6]. Many studies also suggest that drug development on targeting multiple proteins simultaneously can improve efficacy, particularly in the treatment of complex diseases (e.g. cancer and central nervous system disorders) [7,8]. The strategy of pharmaceutical research, defined as the specific binding of a compound to two or more molecular targets, has variously been termed "network-based" discovery, "multi-targeted" drug design, "targeted polypharmacology", or "polypharmacology" [9-17].

One challenge of targeted polypharmacology is to identify the molecular targets that bind the compound. To recognize the proteins with similar binding sites of a given protein sequence or structure, the computational approaches of searching the protein sequence or structure databases are usually utilized [18-21]. As the number of protein structures is increasing, protein structures have been proposed to analyze the structural motifs and to describe the binding environments [22,23]. However, most of these studies [24-28] search for similar local structures or binding sites (active sites) based on only one structural motif. Recently, we have introduced Space-Related Pharmamotif (SRPmotif) method [29] to identify pharma-interfaces (≥ 2 structural motifs) from a set of proteins which share similar binding environments. A pharma-interface is consists of a set of spatially discontinuous pharma-motifs which surround the ligand-binding site. In addition, compounds with similar topology would bind to the same or similar proteins and have similar protein-compound interactions [3,4]. However, few studies focused on exploring the relationships between binding environments and protein-compound interactions. The atomic interactions between a compound and a protein are important when a compound target a protein. The combination of similar binding environments and protein-compound interactions provides the opportunities to explore the molecular binding mechanisms and is helpful for drug repurposing.

To address this issue, we proposed a new concept of "Homopharma" to describe similar binding environments and the relationships of interactions between proteins and compounds. A Homopharma is a set of proteins which have the conserved sub-binding environment at the protein-compound interfaces and a set of compounds with similar topology. Our results demonstrated that protein-compound complexes of a Homopharma perform similar protein-compound interactions and comprise conserved specific residues and important functional sites. According to the Homopharma concept, four similar flavonoid derivatives were tested against 32 human protein kinases using *in vitro *enzymatic profiling. The experimental results identified that the IC_50 _values of 56 and 25 flavonoid-kinase inhibitions are less than 10 μM and 1 μM, respectively. Some novel protein-compound interactions also suggest that these flavonoids could be used as anticancer compounds such as oral and colorectal cancer drugs. These results show that the concept Homopharma is not only useful for identifying potential targets of compounds, but can also reveal the key binding environment. Furthermore, it would be helpful for discovering the new usages for existing drugs. We believe that this approach can be further applied to understand molecular binding mechanisms and take a new direction on drug discovery.

## Materials and Methods

### Homopharma and pharma-interfaces

**Figure 1:**
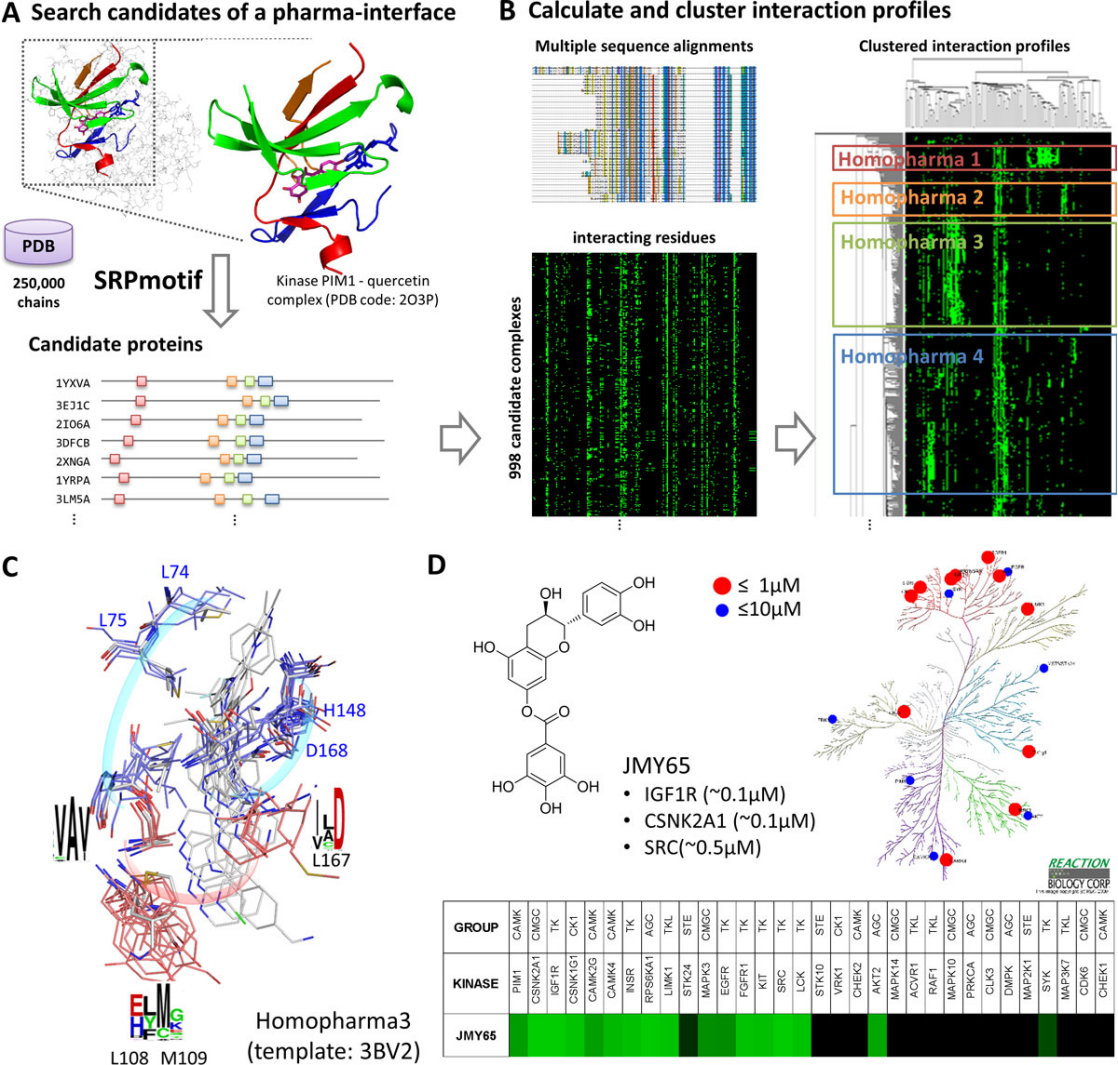
**Overview of identification of Homopharma using serine/threonine-protein kinase Pim-1 and quercetin complex as query**. (A) Search similar pharma-interface candidates of serine/threonine-protein kinase Pim-1 and quercetin complex. (B) The candidates with bound compounds and RMSD ≤ 3Å are used to generate multiple sequence alignment and the protein-compound interaction profile using *i*GEMDOCK. The interaction profile is clustered into several Homopharma groups based on interaction similarity scores. (C) Some superimposed complex structures of Homopharma 3. (D) The flavonoid derivative (JMY65) is tested against 32 selected protein kinases based on Homopharma concept. Among 32 bioassays, the IC_50 _of 11 and 18 kinase-JMY65 interactions are less than 1 μM (red) and 10 μM (blue), respectively. The green and dark denote the inhibition efficiency from high to low.

In this study, we propose a new concept "Homopharma" to describe similar binding environment and the relationships of interactions between proteins and compounds. Proteins sharing similar binding environments can usually be targeted by a set of compound with similar topology. We considered that a Homopharma comprises of a set of proteins which have the conserved sub-binding environment at the protein-compound interfaces and a set of compounds with similar topology. For a complex structure, the proteins with similar pharma-interfaces are first recognized by SRPmotif. The complexes with significant compound topology similarity are reserved. Then, the protein-compound interaction similarity scores are measured. Consequently, the proteins and compounds which share similar protein-compound interactions are considered as a Homopharma. Figure 1 shows the detailed steps to identify a Homopharma from a given complex by the following steps.

#### Step 1: Search candidates of a pharma-interface

SRPmotif is utilized to rapidly search potential targets having similar pharma-interfaces (Figure 1A). A pharma-interface can be described as follows: (1) a pharma-interface is a conserved binding interface of multiple proteins which share similar interfaces and are often inhibited by similar compounds; (2) a pharma-interface consists of a set of spatially discontinuous pharma-motifs; (3) a pharma-motif is a short conserved peptide forming a specific sub-interface with interacting residues and specific physico-chemical properties. Each discontinuous pharma-motif of a query complex is translated to 1D sequence with 23 structural alphabets using κ and α angles defined in the DSSP program [30] and applied to search against Protein Data Bank (PDB) [31] by 3D-BLAST [32,33]. The candidate structures are then superimposed with the discontinuous pharma-motifs of the query complex by DALI [34], which is a structural alignment tool based on contact similarity patterns. The candidate protein-compound complexes of root-mean-square deviation (RMSD) ≤ 3Å are reserved. All search parameters of SRPmotif are default. Accordingly, a pharma-interface which consists of several pharma-motifs is constructed based on the conserved binding interfaces. The complexes of the pharma-interface are then used to estimate the protein-compound interaction profile and to evaluate interaction similarities.

#### Step 2: Calculate protein-compound interaction profiles

A compound would bind to proteins having similar binding environments; as well as proteins often interact with compounds of similar topology. These protein-compound interactions provide the opportunities to explore the molecular binding mechanisms. After identifications of candidate complexes, similarities between all crystal ligands of complexes are calculated by atom pairs (AP) [35]. The structures whose crystal ligands with significant topology similarity (≥ 0.6) to any compound are reserved. To measure the similarity between protein-compound interactions, a multiple protein sequence (or structure) alignment is required. In this work, a multiple protein sequence alignment is utilized and generated by the reserved structures and T-coffee [36]. Finally, the protein-compound interaction profile of candidate complexes is calculated based on *i*GEMDOCK [37]. Different interaction types (electrostatic, hydrogen bonding, and van der Waals) and interactions of main- and side-chain heavy atoms are considered separately.

#### Step 3: Cluster interaction profiles using interaction similarity

After estimations of the protein-compound interaction profile, conserved interaction similarity scores between protein-compound interactions are evaluated. To measure the interacting conservation, the conservation weight of each position of multiple sequence alignment (MSA) is given as:

(1) $$W_{i}^{k}=\frac{f_{i}^{k}}{N}$$

where N is the number of compound members of the pharma-interface and $f_{i}^{k}$ is the number of interactions of the residue in MSA position *i *interacting with compounds with the interaction type *k *(i.e., electrostatic, hydrogen bonding, and van der Waals). The interaction similarity score between two protein-compound interactions (*A *and *B*) is defined as following:

(2) $$S_{c}AB=\frac{\underset{i}{\sum }A_{i}W_{i}\times B_{i}W_{i}}{\underset{i}{\sum }A_{i}{W_{i}}^{2}+{\underset{i}{\sum }B_{i}W_{i}}^{2}-\underset{i}{\sum }A_{i}W_{i}\times B_{i}W_{i}}$$

where *A_i _*and *W_i _*is the interaction and the conservation weight in position *i *of the MSA, respectively.

Consequently, the protein-compound interactions are clustered by two-way hierarchical clustering. These interactions are divided into several Homopharma groups, which consist of similar binding environment, compound topology and interactions.

### Protein-ligand complex validation dataset

To verify the Homopharma concept, protein-compound complexes were obtained from PDB. The complexes are eliminated when compounds are metal ions, surface compounds, cofactors, and small compounds (heavy atoms < 6). Two protein-compound pairs are grouped according to the same proteins or compounds. The protein-compound groups are filtered by number of complexes below 50% of all possible protein-compound combinations. Two groups which overlap with more than 50% protein or compounds are merged. Finally, 176 protein-compound groups that contain 1,325 complex structures including 672 proteins and 471 compounds are used as validation dataset.

## Results and discussion

### Evaluations of compound similarity

A protein-compound complex having significant compound similarity is considered as a candidate of a Homopharma. In order to decide the threshold of compound topology similarity, we collected 3,151 crystal complex structures of 957 proteins. The distribution demonstrates the relationships between percentage and compound topology similarity (Figure S1A in Additional file 1). The precision, recall, and f-measure values were evaluated and the highest F-measure is at 0.36 when the compound topology similarity is 0.6 as well as the precision value is over 0.4. For example, methotrexate (MTX) is the first generation of dihydrofolate reductase (DHFR) inhibitor; the values of compound similarity of COP and DTM to MTX are 0.7 and 0.3, respectively (Figure S1B in Additional file 1). The compound COP is also the first generation of DHFR inhibitor and has similar topology to MTX; however, DTM is the second generation inhibitor and performs a different binding mode. Another compound estrogen (EST) presents similar results. The compound DES (similarity: 0.8) is the estrogen receptor agonist that present the same function with EST, where RAL (similarity: 0.2) is just an estrogen receptor antagonist.

### Evaluations of interaction similarity

**Figure 2:**
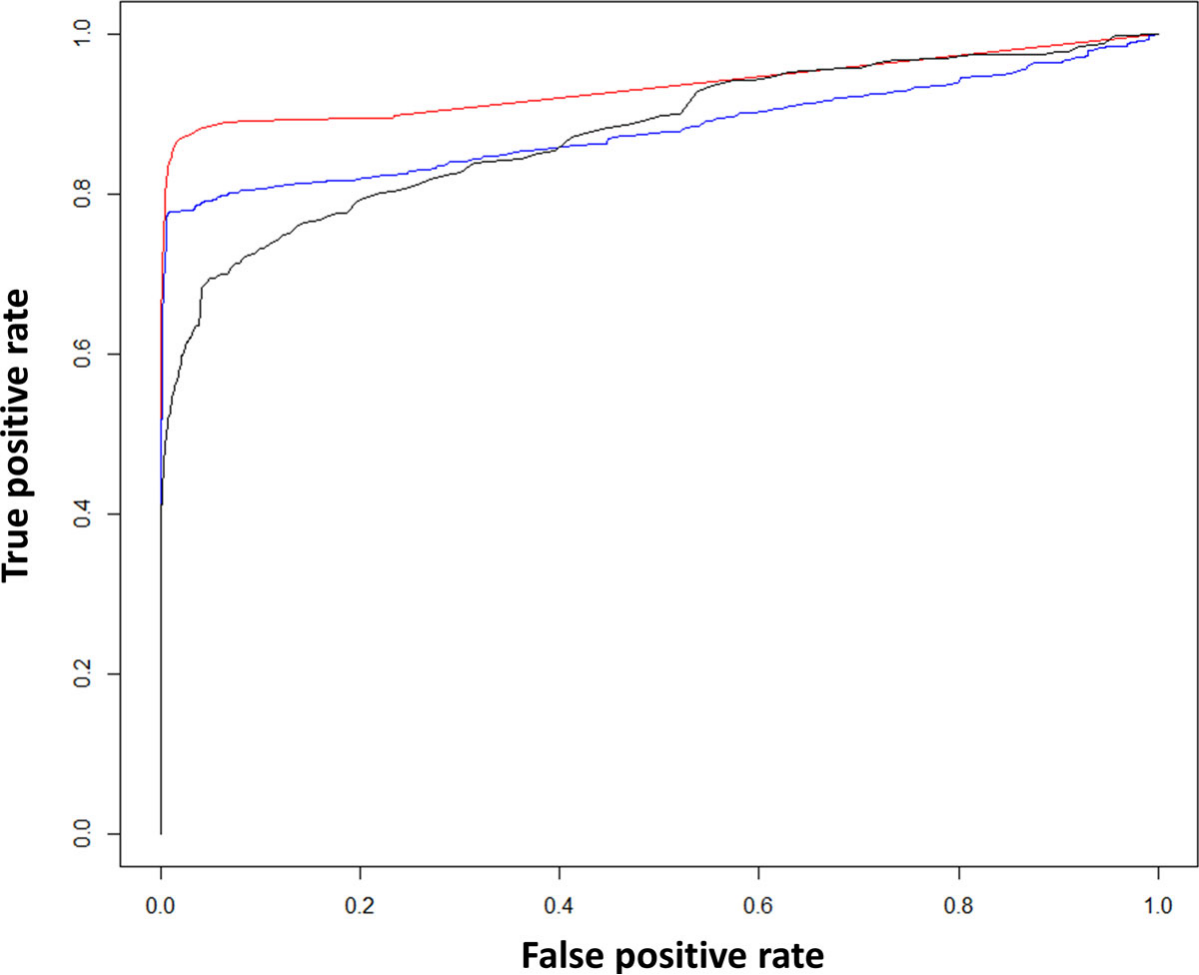
**The receiver operating characteristic curves of different similarities**. The receiver operating characteristic curves using protein similarity (blue), compound similarity (black), and protein-compound interaction similarity (red) based on 176 protein-compound groups.

To evaluate the performance of interaction similarity, we collected 176 protein-compound groups from PDB. Two protein-compound pairs in the same groups are considered as positive pairs; otherwise, pairs are negative pairs. We also evaluated the performance of identifying protein-compound pairs by protein similarity and compound similarity. The thresholds of protein similarity and compound similarity are BLAST e-value ≤ 10^-10 ^and topology similarity ≤ 0.6, respectively. Two pairs are considered as similar interactions while their interaction similarity is greater than 0.6. The receiver operating characteristic (ROC) curves were estimated to compare protein similarity, compound similarity and interaction similarity (Figure 2). The results showed that interaction similarity (red) provides better performance than protein similarity (blue) and compound similarity (black).

### Example analysis: thymidine kinase

**Figure 3:**
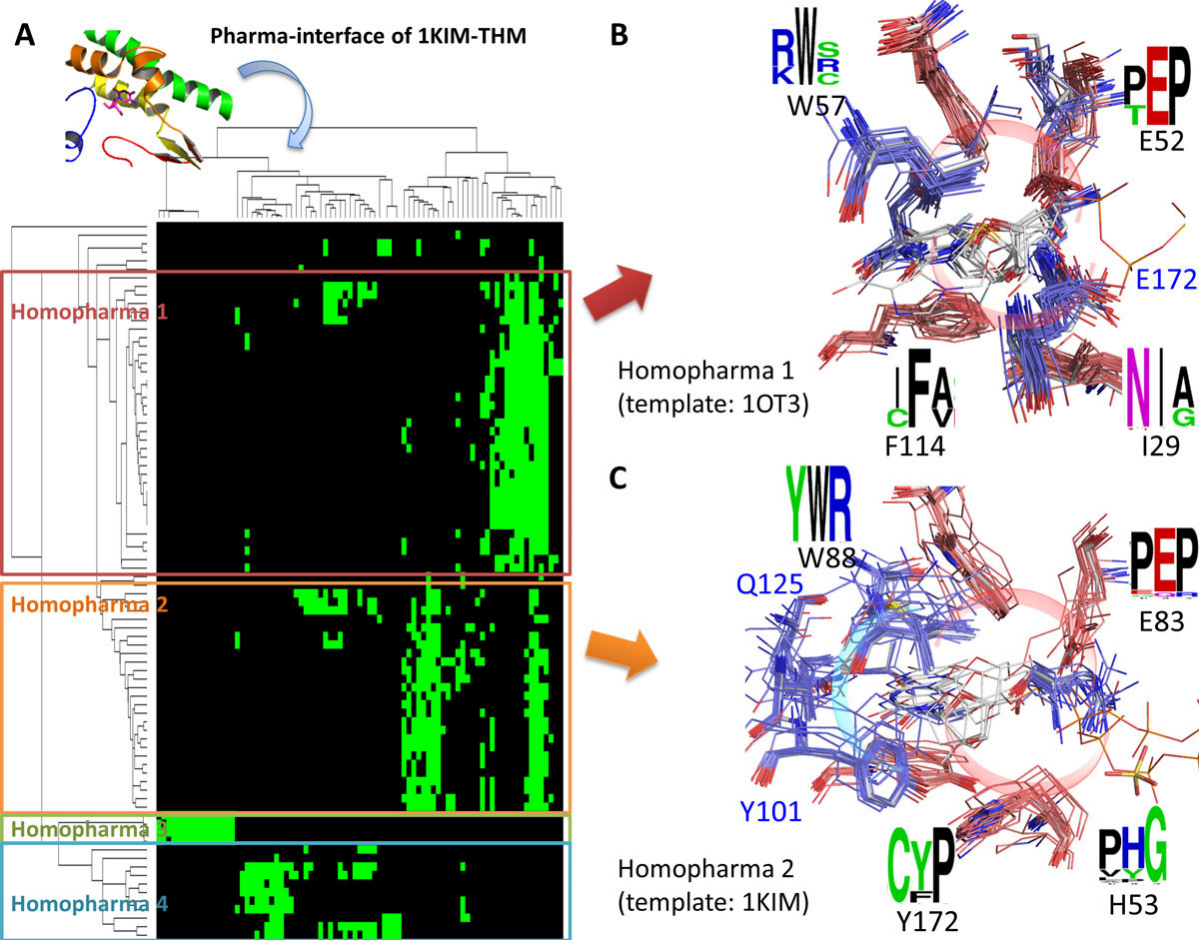
**The pharma-interface and Homopharma groups of thymidine kinase and deoxythymidine complex**. (A) The pharma-interface of thymidine kinase (TK) and deoxythymidine (THM) complex consists of five pharma-motifs. The protein-compound interactions were grouped into four Homopharma groups based on interaction similarity scores. (B) The superimposed structures of Homopharma 1 and conserved interacting residues (residue numbering of PDB code: 1OI3). (C) The superimposed structures of Homopharma 2 and conserved interacting residues (residue numbering of PDB code: 1KIM).

The proteins and compounds in a Homopharma would share conserved interactions and similar physicochemical properties. Therefore, these compounds are often able to inhibit these proteins in a Homopharma. Figure 3A shows that the pharma-interface and Homopharma groups by using the thymidine kinase (TK) of *Herpes simplex virus *and deoxythymidine (THM) complex (PDB code: 1KIM, chain A) as the query complex. Due to specific expression of herpes thymidine kinase (TK) in herpesvirus-infection cells, TK becomes an antiviral target of current drugs, such as acyclovir and vidarabine. The identified pharma-interface contains five conserved pharma-motifs. Furthermore, the clustering of interaction profiles demonstrates protein-compound interactions can be separated into four Homopharma groups.

The complexes in a Homopharma present similar binding modes and have the conserved interacting residues (Figures 3B and 3C). Moreover, the conserved interacting residues of these Homopharma groups are often related to ligand binding and catalytic residues. For example, Figure 3B shows the superimposed structures of Homopharma 1 and conserved interacting residues (residue numbering of PDB code: 1OI3). Several interacting residues (H58, E83, W88, and Y172) are highly conserved in Homopharma 1. Residue E225 is important to form hydrogen bonding that stabilizes the LID region for TK catalytic reaction and H58L/M128F/Y172F triple mutant has also been indicated that related to the resistance of current anti-TK drugs, such as acyclovir [38]. The conserved interaction profiles are presented in Figure S2A in Additional file 1. However, Homopharma 2 has different conserved interacting residues to Homopharma 1 (Figures 3C and S2B in Additional file 1). Residues H58, E83, W88, and Y172 are also conserved in Homopharma 1 as well; however, the specific conserved residue R222 of Homopharma 2 interacts with phosphate group of TK substrates [38]. Furthermore, Homopharma 3 and 4 also present different binding environments (Figures S2C and S2D in Additional file 1).

### Example analysis: protein kinases and flavonoid derivatives

Flavonoids are a class of plant secondary metabolites and have more than 5,000 kinds of derivatives. Flavonoid derivatives have been crystallized within more than 300 protein complex structures, such as protein kinases. Protein kinases play important roles in cell growth and signal transduction [39,40]. Protein kinases are one of the most important classes of drug targets, because the deregulation of kinase functions is often implicated in many diseases, such as cancers and neurological and metabolic diseases [40-42]. Although protein kinases share a highly conserved ATP binding environment; however, identical kinase inhibitor has varied inhibitory effects on different kinases [3,4]. Because protein kinases are involved in many complex diseases, many flavonoid derivatives whose biological activities are known on kinases may be repositioned to potential candidates of other diseases.

**Figure 4:**
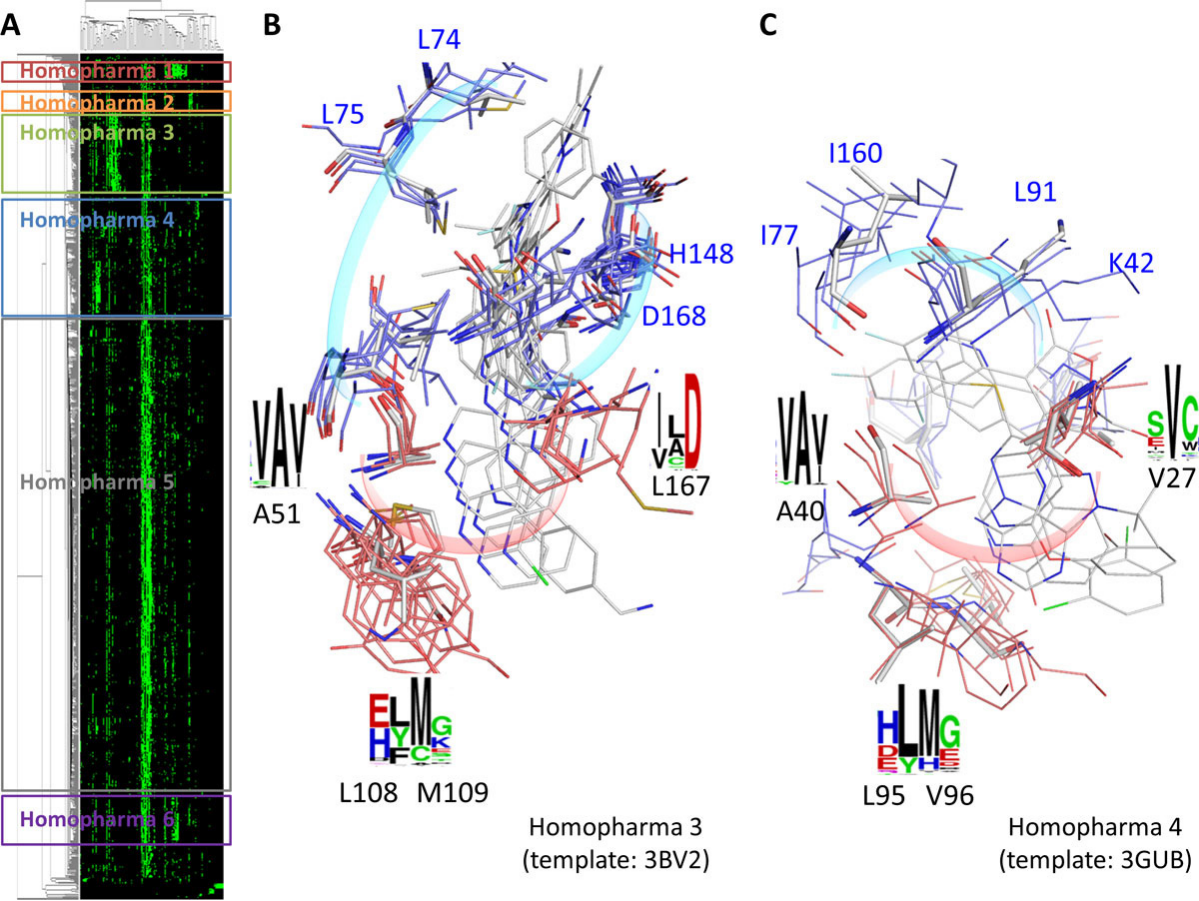
**The pharma-interface and Homopharma groups of serine/threonine-protein kinase Pim-1 and quercetin complex**. (A) The pharma-interface of serine/threonine-protein kinase Pim-1 (PIM1) and quercetin complex consists of four pharma-motifs. The protein-compound interactions are grouped into six Homopharma groups. (B) The superimposed structures of Homopharma 3 and conserved interacting residues (residue numbering of PDB code: 3BV2). (C) The superimposed structures of Homopharma 4 and conserved interacting residues (residue numbering of PDB code: 3GUB).

In this study, we use serine/threonine-protein kinase Pim-1 (PIM1) and quercetin complex (PDB code: 2O3P, chain A) to identify pharma-interface and Homopharma groups. After recognitions of 975 protein-compound complexes by four pharma-motifs, six Homopharma groups are identified according to the interaction similarity (Figures 4A and S3 in Additional file 1). Among these groups, Homopharma 3 shows the highly conserved interactions to DFG motif [43], which is the beginning of activation loop of kinase (Figure 4B). The conformation of DFG motif is relevant to kinase activation and the conserved aspartic acid binds directly to the magnesium ion cofactor orienting the γ-phosphate of ATP for transfer. Conserved interacting residues, such as L108 and M109 (residue numbering of PDB code: 3BV2), are involved in hinge motif, which interacts with adenine of ATP. Most compounds of Homopharma 3 belong to Type II kinase inhibitors, which occupy the ATP binding region and allosteric pocket exposed in the kinase inactive conformations [44,45].

Homopharma 4 also performs the conserved interactions to hinge motif (Figure 4C). However, these compounds of Homopharma 4 belong to Type I kinase inhibitors, which target kinases with active conformation and directly compete with ATP binding. Furthermore, compounds of Homopharma 2 are also Type I kinase inhibitors, but have different topology and interacting residues (Figure S3B in Additional file 1). These compounds perform more interactions with residues of C-lobe pocket. Previous study showed that the conserved aspartic acid in C-loop is important in positioning the substrate hydroxyl for in-line nucleophilic attack [46]. Similar observations can be found in other Homopharma groups (Figure S3 in Additional file 1).

### Kinase profiling experiment results

**Figure 5:**
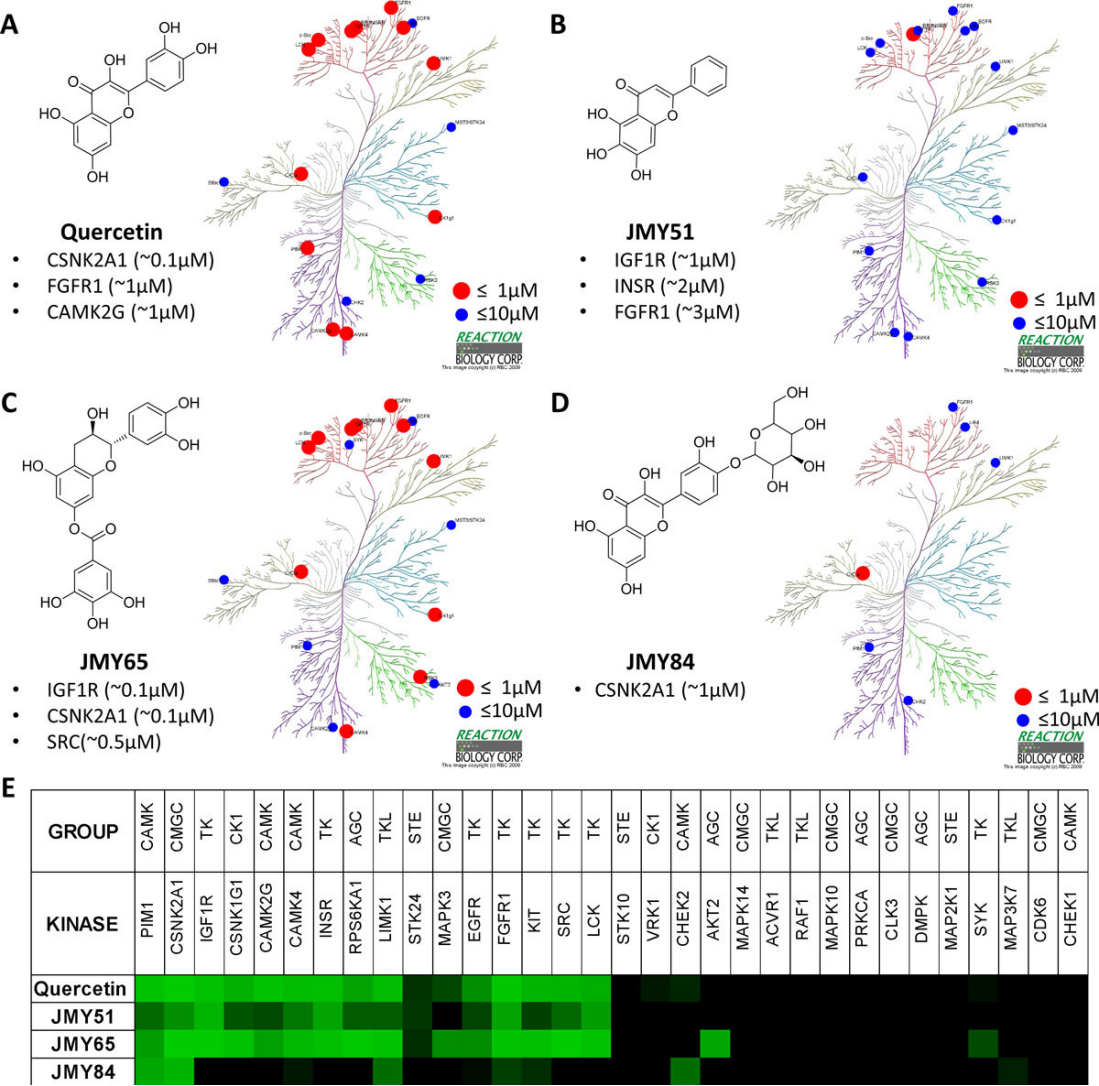
**The *in vitro *enzymatic profiling results**. (A) Quercetin and its target kinases. (B) JMY51 and its target kinases. (C) JMY65 and its target kinases. (D) JMY84 and its target kinases. The red and blue points mean the bioactivities are ≤ 1 μM and ≤ 10 μM, respectively. Kinome tree representation is prepared using Kinome Mapper (http://www.reactionbiology.com/apps/kinome/mapper/LaunchKinome.htm). (E) The *in vitro *enzymatic profiling results of 32 protein kinases and four flavonoid derivatives. The green and dark denote the inhibition efficiency from high to low.

To examine the ability of Homopharma for discovering novel potential targets, we selected quercetin and three similar flavonoid derivatives to test potential target kinases in Homopharma groups. Four selected flavonoid derivatives were obtained from Toronto Research Chemicals (Figure 5). Based on six Homopharma groups of the PIM1-quercetin complex, test kinases were predicted as potential targets by the following step: (1) four flavonoids were docked to these kinase structures of the identified pharma-interface by *i*GEMDOCK; (2) interaction profiles were analyzed from the docking poses that have the lowest energy; (3) interaction similarity scores were measured to the PIM1-quercetin complex; (4) the kinases of the same Homopharma group have the priority with selection. Most kinases were selected from Homopharma 2, 4, and 5. Furthermore, some kinases which are either not considered potential targets or not searched by pharma-interface were also collected. Eventually, 32 protein kinases were chosen (Table 1 and Figure S4 in Additional file 1).

**Table 1:** List of 32 tested protein kinases for *in vitro *enzymatic profiling.

Sequence group	Symbol name	Kinase SK number	Kinase description
AKT2	AGC	SK019	v-akt murine thymoma viral oncogene homolog 2
DMPK	AGC	SK111	dystrophia myotonica-protein kinase
PRKCA	AGC	SK303	protein kinase C, alpha
RPS6KA1	AGC	SK338	ribosomal protein S6 kinase, 90kDa, polypeptide 1
CAMK2G	CAMK	SK060	calcium/calmodulin-dependent protein kinase (CaM kinase) II gamma
CAMK4	CAMK	SK061	calcium/calmodulin-dependent protein kinase IV
CHEK1	CAMK	SK078	CHK1 checkpoint homolog (S. pombe)
CHEK2	CAMK	SK079	CHK2 checkpoint homolog (S. pombe)
PIM1	CAMK	SK291	pim-1 oncogene
CSNK1G1	CK1	SK647	casein kinase 1, gamma 1
VRK1	CK1	SK389	vaccinia related kinase 1
CDK6	CMGC	SK071	cyclin-dependent kinase 6
CLK3	CMGC	SK092	CDC-like kinase 3
CSNK2A1	CMGC	SK088	casein kinase 2, alpha 1 polypeptide
MAPK10	CMGC	SK190	mitogen-activated protein kinase 10
MAPK14	CMGC	SK264	mitogen-activated protein kinase 14
MAPK3	CMGC	SK134	mitogen-activated protein kinase 3
MAP2K1	STE	SK217	mitogen-activated protein kinase kinase 1
STK10	STE	SK426	serine/threonine kinase 10
STK24	STE	SK246	serine/threonine kinase 24 (STE20 homolog, yeast)
EGFR	TK	SK118	epidermal growth factor receptor (erythroblastic leukemia viral (v-erb-b) oncogene homolog, avian)
FGFR1	TK	SK143	fibroblast growth factor receptor 1 (fms-related tyrosine kinase 2, Pfeiffer syndrome)
IGF1R	TK	SK174	insulin-like growth factor 1 receptor
INSR	TK	SK178	insulin receptor
KIT	TK	SK201	v-kit Hardy-Zuckerman 4 feline sarcoma viral oncogene homolog
LCK	TK	SK206	lymphocyte-specific protein tyrosine kinase
SRC	TK	SK357	v-src sarcoma (Schmidt-Ruppin A-2) viral oncogene homolog (avian)
SYK	TK	SK363	spleen tyrosine kinase
ACVR1	TKL	SK026	activin A receptor, type I
LIMK1	TKL	SK412	LIM domain kinase 1
MAP3K7	TKL	SK364	mitogen-activated protein kinase kinase kinase 7
RAF1	TKL	SK324	v-raf-1 murine leukemia viral oncogene homolog 1

*In vitro *enzymatic profiling of the 32 member kinase panel was performed at Reaction Biology Corporation (http://www.reactionbiology.com, Malvern, PA) using the "HotSpot" assay platform [3]. Each kinase activity assay was performed in duplicate with inhibitor concentration of 10 μM and an ATP concentration of 10 μM. The experimental results show that three similar flavonoids (quercetin, JMY51, and JMY65) have similar inhibitory effects on the tested 32 kinases. The results indicate that kinases in the same Homopharma group are inhibited by similar compounds. Our results also discover novel interactions, for example, JMY65 has SRC, LCK, and KIT inhibitory activities (Figures 5C and 5E). Moreover, JMY65 could be used to treat oral cancer because of its inhibitory effects on AKT2, IGF1R, EGFR, and MAPK3 (Figures 5C and 5E), which are highly expressed in oral cancer cells. Another compound JMY84 could also be used as drug candidates of colorectal cancer due to its effects on CHEK2, LIMK1, and FGFR1 (Figures 5D and 5E).

**Figure 6:**
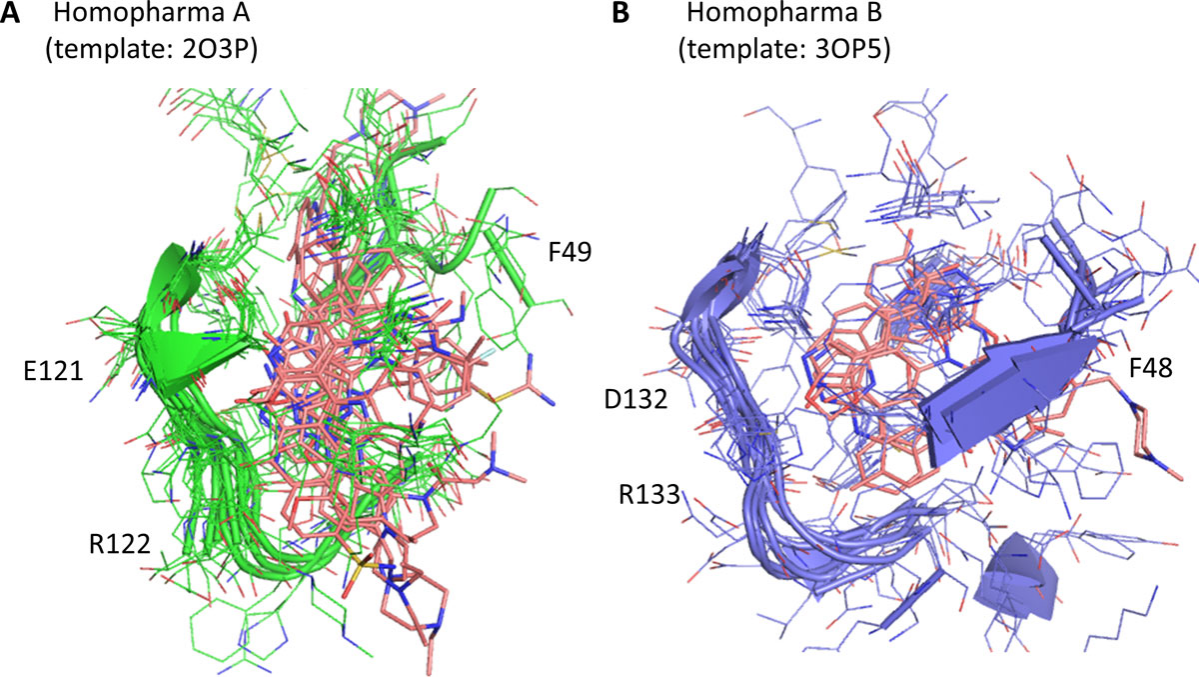
**The superimposed structures and enzymatic profiling results**. (A) The 16 protein kinases inhibited by four flavonoids (quercetin, JMY51, JMY65, and JMY84) are superimposed (residue numbering of PDB code: 2O3P). Kinases and flavonoids are colored by green and pink, respectively. (B) The structures of 16 protein kinases, which are not inhibited by four flavonoids, are superimposed (residue numbering of PDB code: 3OP5). Kinases are colored by blue.

These 32 tested kinases were divided into two Homopharma groups and the superimposed structures of two groups are presented in Figure 6. Most kinases of Homopharma A are inhibited by tested flavonoid derivatives and have different interacting residues and binding environments than Homopharma B. The locations of DFG motif are varied between these groups (Figure 6A). The co-crystal compounds also suggest the difference of binding environments. These results showed that our approach could discover potential targets of a set of similar compounds based on Homopharma group.

## Conclusions

In this study, we propose a new concept of "Homopharma", combining similar binding environments and protein-compound interaction profiles, to explore the molecular binding mechanisms and drug repurposing. A Homopharma consists of a set of proteins sharing conserved binding environment and a set of compounds which have similar structures and functional groups. In a Homopharma, the compounds are often able to inhibit these proteins because their interactions and physicochemical properties are often consensus. Experimental results show that protein-compound complexes of a Homopharma often perform similar interactions in which formed by conserved binding residues (protein sites) and similar important functional groups (compound sites). According to the Homopharma concept, we successfully discovered 56 flavonoid-kinase inhibitions (IC_50 _≤ 10 μM) by *in vitro *enzymatic profiling. Among these 56 inhibitions, the IC_50 _values of 25 bioassays are less than 1 μM and these flavonoids can be considered as potential anticancer compounds. These results show that Homopharma is not only useful to identify potential targets of compounds, but also can reveal the key binding environments. We believe that the Homopharma concept is useful for understanding molecular binding mechanisms and providing new opportunities for drug repurposing.

## Competing interests

The authors declare that they have no competing interests.

## Authors' contributions

YYC, CTL, and JMY conceived and designed the experiments. YYC, JHT, KHL, and JMY implemented the materials/analysis programs. YYC, JHT, KHL, CTL, KCH, and JMY performed the experiments and analysed the data. YYC, JHT, KHL, CTL, and JMY wrote the paper.

## Supplementary Material

Additional file 1 Click here for file

## Declarations

The publication costs for this paper were funded by the Taiwan Ministry of Science and Technology grant MOST103-2113-M-009-010 awarded to JMY.

This article has been published as part of *BMC Genomics *Volume 15 Supplement 9, 2014: Thirteenth International Conference on Bioinformatics (InCoB2014): Computational Biology. The full contents of the supplement are available online at http://www.biomedcentral.com/bmcgenomics/supplements/15/S9.
