# Supplementary material for: Homopharma: A new concept for exploring the molecular binding mechanisms and drug repurposing
Source: BMC Genomics. 2014 Dec 8;15(Suppl 9):S8. doi: 10.1186/1471-2164-15-S9-S8 (PMC4290623; doi:10.1186/1471-2164-15-S9-S8)
Supplement: Additional file 1 [file 1471-2164-15-S9-S8-S1.pdf]

Additional files

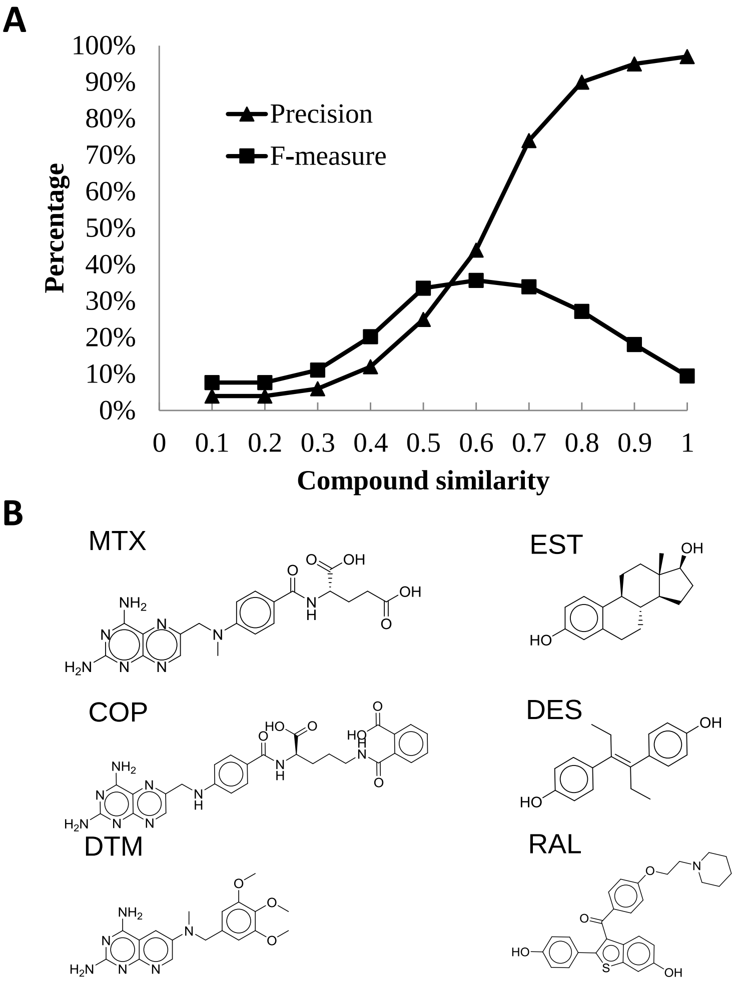

Supplementary Figure S1 – The relationship of precision values and f-measure between the percentage and compound similarity.

(A) The relationship between F-measure and compound similarity. (B) Methotrexate (MTX) is one of DHFR first generation inhibitors. The compound similarity scores of COP and DTM to MTX are 0.78 and 0.36, respectively. Estrogen (EST) is the ER agonist. The compound similarity score of DES and RAL to EST are 0.62 and 0.27, respectively.

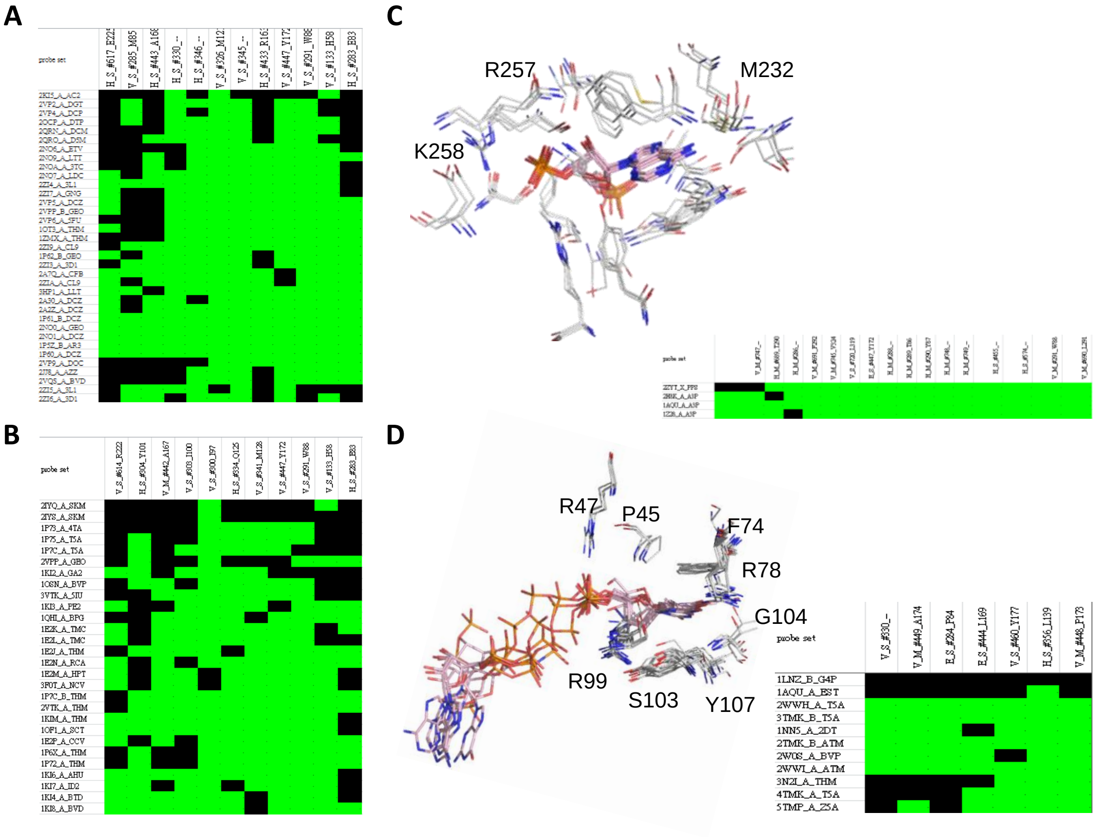

Supplementary Figure S2 - The interaction profiles and superimposed structures of thymidine kinase and deoxythymidine complex.

(A) The conserved interacting residues of Homopharma 1 of thymidine kinase (TK) and deoxythymidine (THM) complex. (B) The conserved interacting residues of Homopharma 2 of TK and THM complex. (C) The superimposed structures of Homopharma 3 and conserved interacting residues. (D) The superimposed structures of Homopharma 4 and conserved interacting residues.

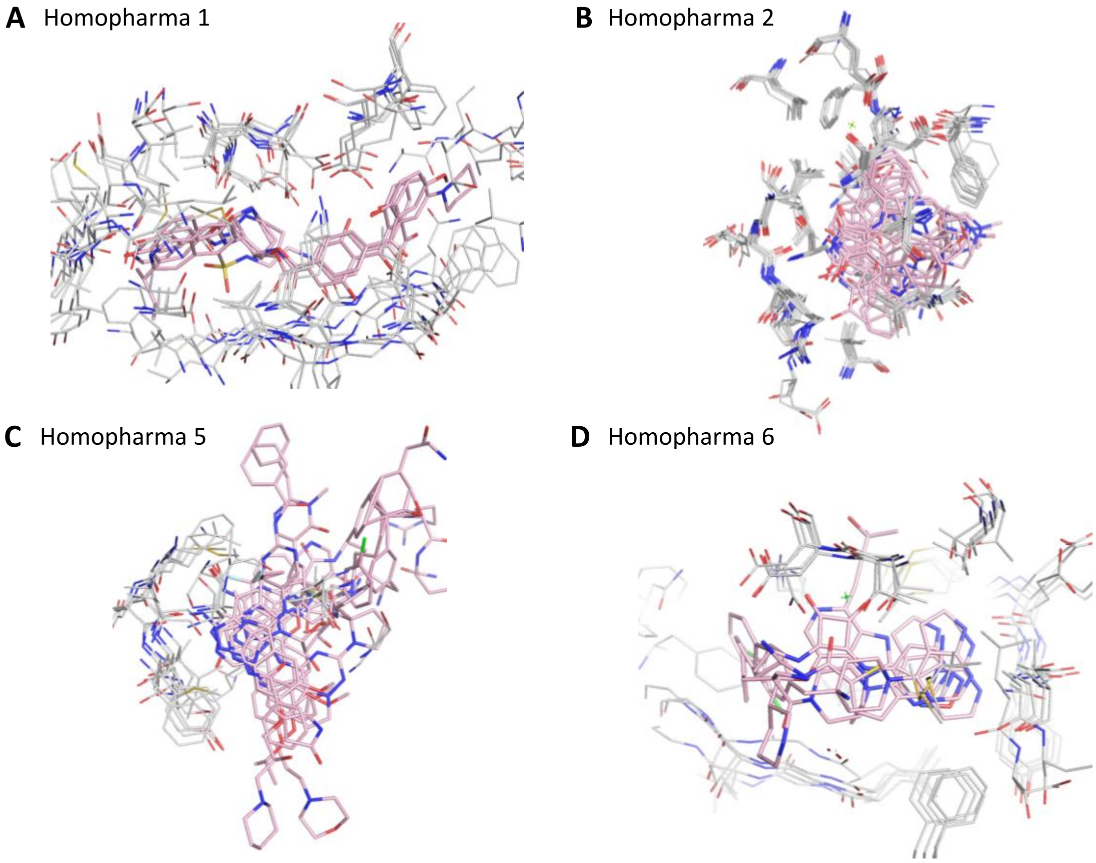

Supplementary Figure S3 - The superimposed structures of serine/threonine-protein kinase Pim-1 and quercetin complex.

(A) The superimposed structures of Homopharma 1 and conserved interacting residues. (B) The superimposed structures of Homopharma 2 and conserved interacting residues. (C) The superimposed structures of Homopharma 5 and conserved interacting residues. (D) The superimposed structures of Homopharma 6 and conserved interacting residues.

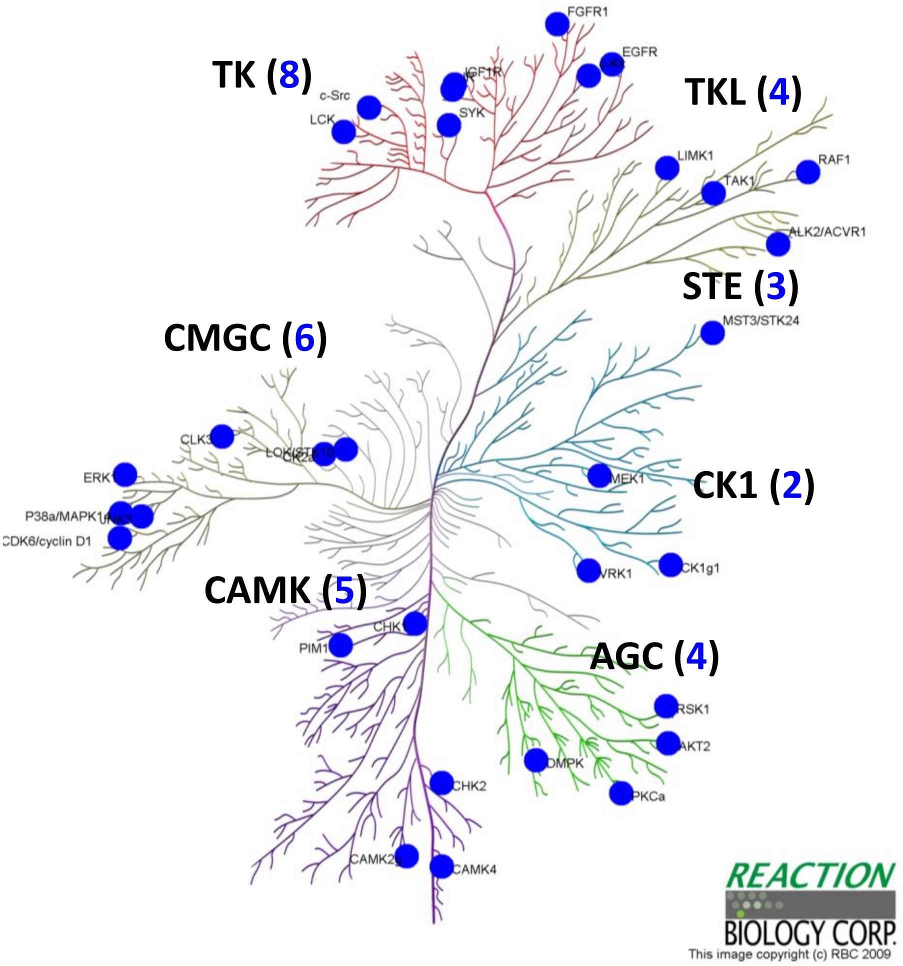

Supplementary Figure S4 - The 32 tested protein kinases of *in vitro* enzymatic profiling.

The 32 tested protein kinases of in vitro enzymatic profiling. Kinome tree representation was prepared using Kinome Mapper (http://www.reactionbiology.com/apps/kinome/mapper/LaunchKinome.htm).
